# Raman Micro­spectroscopy and Imaging of Filamentous Fungi

**DOI:** 10.1264/jsme2.ME22006

**Published:** 2022-04-07

**Authors:** Shinsuke Shigeto, Norio Takeshita

**Affiliations:** 1 Department of Chemistry, School of Science, Kwansei Gakuin University, Sanda, Hyogo 669–1337, Japan; 2 Microbiology Research Center for Sustainability (MiCS), Faculty of Life and Environmental Sciences, University of Tsukuba, Ibaraki, Tsukuba 305–8572, Japan

**Keywords:** Raman, microspectroscopy, Filamentous Fungi, imaging, Fluorescence imaging

## Abstract

Filamentous fungi grow by the elongation of tubular cells called hyphae and form mycelia through repeated hyphal tip growth and branching. Since hyphal growth is closely related to the ability to secrete large amounts of enzymes or invade host cells, a more detailed understanding and the control of its growth are important in fungal biotechnology, ecology, and pathogenesis. Previous studies using fluorescence imaging revealed many of the molecular mechanisms involved in hyphal growth. Raman microspectroscopy and imaging methods are now attracting increasing attention as powerful alternatives due to their high chemical specificity and label-free, non-destructive properties. Spatially resolved information on the relative abundance, structure, and chemical state of multiple intracellular components may be simultaneously obtained. Although Raman studies on filamentous fungi are still limited, this review introduces recent findings from Raman studies on filamentous fungi and discusses their potential use in the future.

## Filamentous fungi

Filamentous fungi grow by the elongation of tube-like cells known as hyphae; the extension and branching of hyphal tips result in the formation of a hyphal network called the mycelium (Riquelme *et al.*, 2018). In the process of extending hyphae, fungi secrete various extracellular enzymes that break down organic matter in the surrounding environment, which is then absorbed by fungi as nutrients. In nature, filamentous fungi play a vital role as decomposers and are essential for the recycling of organic material (Treseder and Lennon, 2015; Perez *et al.*, 2020). Filamentous fungi include species such as kōji (*Aspergillus oryzae*), which are used in the production of traditional fermented food, including sake, soy sauce, and miso (Takeshita, 2020; Yasui *et al.*, 2020). In recent years, filamentous fungi have been used in the bioindustry to produce organic acids, useful enzymes, and antibiotics (Meyer *et al.*, 2016). Hyphal invasive growth into host cells (plant or animal) is essential for pathogenicity and symbiosis (Rodriguez *et al.*, 2009; Gow *et al.*, 2011; Dean *et al.*, 2012; Garcia-Vidal *et al.*, 2013). The high capacity of filamentous fungi to secrete several enzymes and their invasive growth into host cells are closely related to mycelial growth and are important for fungal biotechnology, ecology, and pathogenicity.

## Fluorescence imaging

Advances in genetic manipulation and fluorescence microscopy over the past decade have allowed for the real-time tracking of the dynamics of fluorescent fusion proteins in fungal hyphae. Previous studies using fluorescence imaging revealed the elaborate molecular machinery of polarized tip growth, particularly in the model filamentous fungi *Aspergillus nidulans* and *Neurospora crassa* (Fischer *et al.*, 2008; Takeshita, 2016; Takeshita *et al.*, 2017; Riquelme *et al.*, 2018). This machinery includes the orchestrated actions of the actin and microtubule cytoskeletons with motor proteins (Berepiki *et al.*, 2011; Egan *et al.*, 2012; Takeshita *et al.*, 2014), secretory vesicle accumulation, the resulting formation of the Spitzenkörper (Taheri-Talesh *et al.*, 2008; Riquelme and Sánchez-León, 2014), and exocytosis and endocytosis (Shaw *et al.*, 2011; Pinar and Peñalva, 2021).

Super-resolution imaging and high-speed imaging have provided important information on the maintenance of polarity and exocytosis (Ishitsuka *et al.*, 2015; Zhou *et al.*, 2018), and novel insights into hyphal growth as a dynamic system (Takeshita *et al.*, 2017; Takeshita, 2018). A live imaging ana­lysis revealed correlations among intracellular Ca^2+^ levels, actin assembly, and exocytosis in growing hyphal tips (Takeshita *et al.*, 2017). Collectively, these findings indicate that pulsed Ca^2+^ influxes coordinate the temporal control of actin assembly and exocytosis, which results in stepwise cell extension.

Nevertheless, difficulties are still associated with simultaneously examining the distribution of different types of biomolecules and their chemical information in mycelial cells. This limitation has hindered our holistic understanding of the fungal mycelium as a multicellular system. Therefore, alternative approaches are needed to acquire detailed chemical information that is challenging to obtain by conventional fluorescence imaging.

## Raman microspectroscopy

Due to their high chemical specificity and label-free, non-destructive properties, Raman microspectroscopy and imaging methods provide a powerful alternative for *in vivo* studies of prokaryotic cells (Schuster *et al.*, 2000; Xie and Li, 2003; Noothalapati Venkata *et al.*, 2011; Li *et al.*, 2012; Zheng *et al.*, 2013) and eukaryotic cells, including yeast (Huang *et al.*, 2005; Singh *et al.*, 2006; Huang *et al.*, 2011; Noothalapati *et al.*, 2016) and human cells (Puppels *et al.*, 1990; Uzunbajakava *et al.*, 2003; Okada *et al.*, 2012; El-Mashtoly *et al.*, 2014). Simultaneous space-resolved information is provided on the relative abundance, structure, and chemical state of multiple intracellular components. Raman microspectroscopy is based on the Raman effect, which is the inelastic scattering of photons by a molecule (Raman and Krishnan, 1928). When a molecule is irradiated with light, scattering occurs at a frequency that is shifted by a molecular vibrational frequency with respect to the incident light as a result of the light–molecule interaction. This Raman scattered light is many orders of magnitude weaker in intensity than fluorescence, but provides “molecular fingerprints” in the form of Raman spectra.

While the growth of a hypha occurs at the hyphal tip, branching may result in newly growing tips along the hypha. A single hypha is composed of several spatially separated distinct regions, each of which exhibits different physiological activities and functions. The mycelium, which is assumed to exhibit heterogeneous bioactivity in each region, is a suitable target for a Raman ana­lysis; however, Raman studies on filamentous fungi are still limited. The spectral features of crystalline mannitol, a fungal polyol with a complex protective role, were elucidated by Raman spectroscopy on the hyphal composition of *Curvularia protuberata*, which showed the irregular distribution of mannitol in the mycelium (Isenor *et al.*, 2010). The distribution of cytochromes in the mycelium of the model basidiomycete, *Schizophyllum commune*, was revealed by Raman spectroscopy in combination with its non-linear variant (coherent anti-Stokes Raman scattering) (Walter *et al.*, 2010). Based on Raman images of cytochrome marker bands, mitochondrial activity, which may be indirectly probed by cytochrome concentrations, was found to be higher in the apical and branching parts of the mycelium than in the older parts. Despite earlier work, the large amount of information available from Raman spectroscopic data has not yet been fully exploited to visualize the spatial distribution of major intracellular components in these mycelia.

## Chemical distribution and dynamics by Raman

**Fig. 1.**
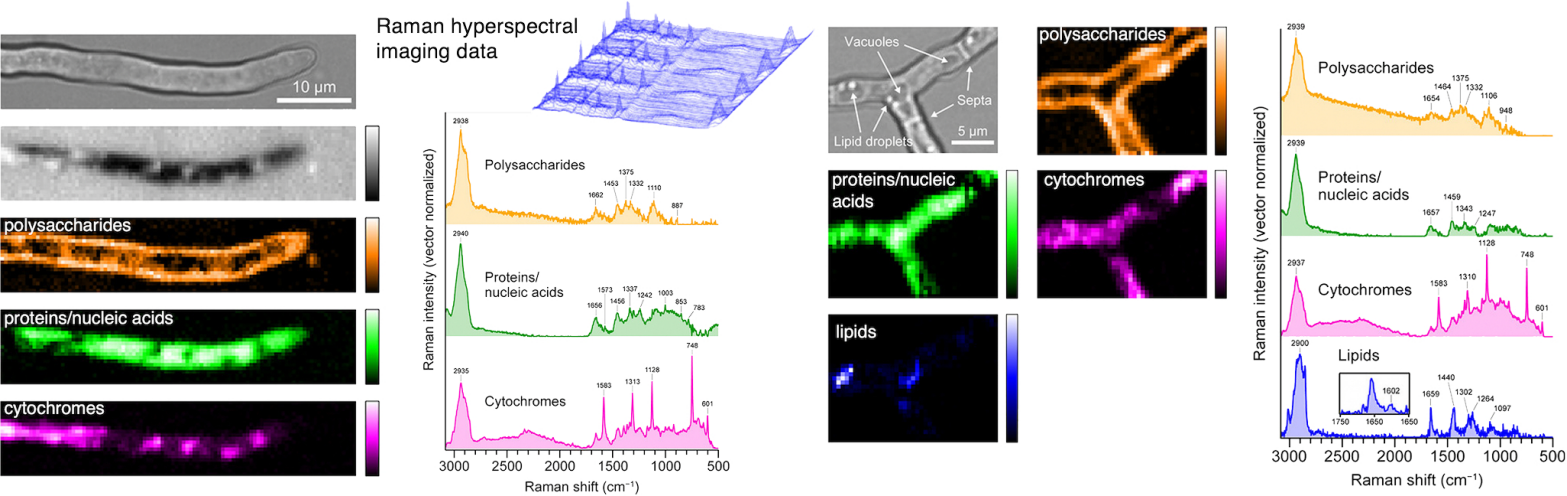
MCR–ALS Raman imaging of a hyphal tip region (left) and branching region (right) of *Aspergillus nidulans*. Spatial distribution (Raman images) and intrinsic Raman spectra of components derived from the MCR–ALS ana­lysis: polysaccharides (yellow), proteins/nucleic acids (green), cytochromes (magenta), and lipids (blue). The CCD exposure time was 1‍ ‍s pixel^–1^; therefore, the total time required to obtain images was ~40‍ ‍min for the tip region (left) and ~24‍ ‍min for the branching region (right). Data were reproduced with permission from Yasuda *et al.*, 2019. Copyright 2019 American Chemical Society.

We recently reported the extraction of both spatial and chemical information on polysaccharides, lipids, and proteins, including cytochromes, in different characteristic regions of the model fungus *A. nidulans* (Fig. 1, Yasuda *et al.*, 2019). This was achieved by a multivariate curve resolution–alternating least squares (MCR-ALS) ana­lysis on Raman hyperspectral imaging data. MCR-ALS, a multivariate data ana­lysis technique, decomposes hyperspectral imaging data into the product of two low-rank matrices consisting of several major components under non-negative constraints (Paatero and Tapper, 1994; Lee and Seung, 1999). This analytical method was applied to study the cell division dynamics of the unicellular fungus *Schizosaccharomyces pombe* (Huang *et al.*, 2012). MCR-ALS Raman imaging of the apical, basal, and lateral branching regions of the mycelium of *A. nidulans* was performed, and the heterogeneous distribution of major subcellular components was consistently visualized in all mycelial segments. In addition, the MCR-ALS ana­lysis provided the molecular fingerprints of these components, which also revealed *in vivo* chemical properties, such as the cell wall composition and the types and redox states of cytochromes (reduced and oxidized forms).

Nanoscale secondary ion mass spectrometry combined with stable isotope probing (SIP) measures the distribution of stable isotopes with high spatial resolution below 50‍ ‍nm and has been utilized in biological research (Musat *et al.*, 2012); however, it is a destructive technique by nature. Raman spectroscopy is a promising tool for *in vivo* metabolic imaging when combined with SIP methods because it allows us to view molecular vibrations in all forms of microscopic samples in a non-destructive manner. In addition to being non-destructive, SIP provides Raman microspectroscopy with the ability to probe the enrichment of stable isotopes (such as ^2^H and ^13^C) in chemical bonds (Haider *et al.*, 2010; Noothalapati Venkata and Shigeto, 2012; Berry *et al.*, 2015). This is because the substitution of heavier isotopes reduces the frequency of the vibrational modes associated with the bond. The magnitude of isotope downshifts may be large, moderate, or virtually undetectable depending on the vibrational mode (such as stretching, bending, and deformation). The intensity of these isotope-shifted bands is a quantitative indicator of the extent to which stable isotopes are enriched.

**Fig. 2.**
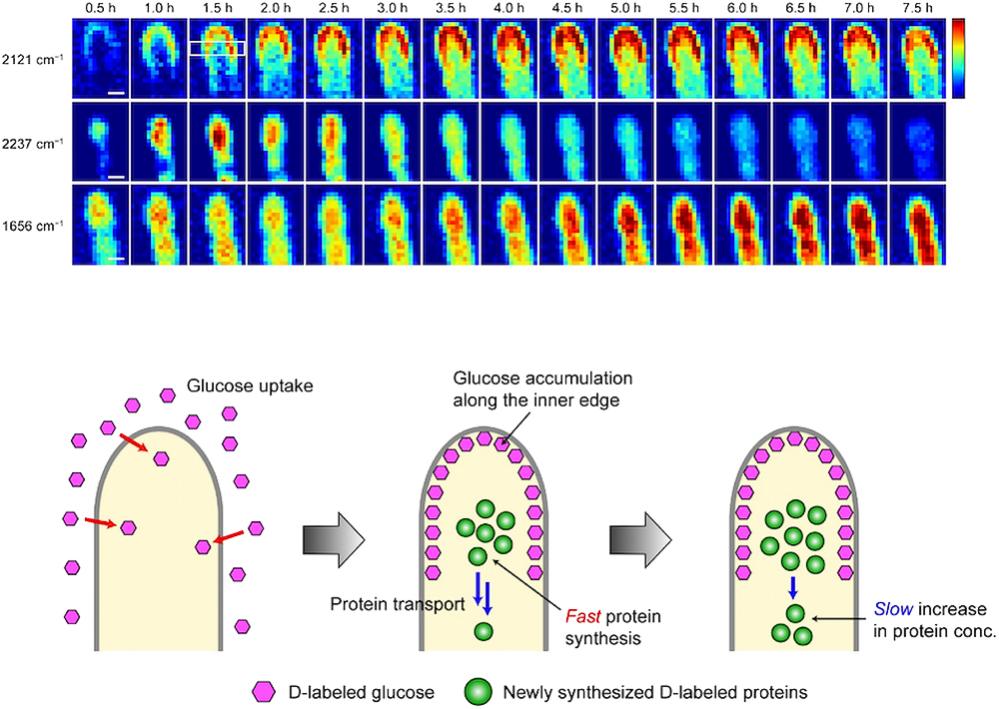
Time-lapse deuterium-labeled Raman imaging of the *Aspergillus nidulans* hyphal tip. Time-lapse Raman images of three deconvolved bands in the C–D stretching region at 2,121 and 2,237‍ ‍cm^–1^ and Raman images of the 1,656‍ ‍cm^–1^ bands. The CCD exposure time was 1‍ ‍s pixel^–1^; therefore, the total time required to obtain each time-lapse image was ~5‍ ‍min. An image showing glucose uptake/accumulation and protein synthesis/transport processes in an *A. nidulans* hypha. Data were reproduced with permission from Yasuda *et al.*, 2021.

Time-lapse Raman imaging using a deuterium (D) tracer visualized spatiotemporally varying metabolic activity within the hyphal tip of *A. nidulans* (Yasuda *et al.*, 2021). By analyzing the carbon–deuterium (C–D) stretching Raman band with spectral deconvolution, we visualized glucose accumulation along the inner edge of the hyphal tip and the synthesis of new proteins from D-labeled glucose specifically taken up at the central part of the apical region (Fig. 2). The signal at the inner edge of the hyphal tip appeared to an active endocytosis zone (Araujo-Bazán *et al.*, 2008). D-labeled glucose is anabolically incorporated into proteins near the center of the hyphal apical region. As newly synthesized proteins near the tip are gradually diffused and transported, the protein concentration behind the tip increases. D-labeled Raman imaging offers a broadly applicable platform for the study of metabolic dynamics in filamentous fungi.

## Raman and secondary metabolites

Filamentous fungi have served as repositories of bioactive secondary metabolites that form the backbone of many existing drugs. *Penicillium chrysogenum* produces β-lactam antibiotics including penicillin G (Weber *et al.*, 2012). A MCR-ALS ana­lysis of Raman spectra obtained from *P. chrysogenum* cells revealed the distribution of several components, including proteins and lipids, in the mycelium (Horii *et al.*, 2020). In addition, the subcellular localization of penicillin G may be observed because the penicillin G component may be separated by a MCR-ALS ana­lysis using the Raman spectra of penicillin G recorded from a standard sample. Penicillin G was discretely distributed in the mycelium and localized in a particulate form. A previous study suggested that penicillin G was biosynthesized inside peroxisomes (Kurzątkowski *et al.*, 2014). This method is the first example of the direct visualization of penicillin G in the mycelium and may be used to screen for the microbial production of bioactive substances. In a subsequent study, this group provided evidence from a Raman-MCR ana­lysis to suggest that extracellular vesicles (EVs) were involved in the release of penicillin G from *P. chrysogenum* cells (Samuel, A.Z., *et al.* 2021. Raman microspectroscopy imaging ana­lysis of extracellular vesicles (EVs) biogenesis by filamentous fungus *Penicilium chrysogenum*. *bioRxiv* 2021.11.04.467387; doi: https://doi.org/10.1101/2021.11.04.467387).

## Raman and genetics

To identify or biologically characterize fungi by Raman spectroscopy, the molecular origin of the Raman response needs to be identified and background fluorescence must be separated. Background fluorescence may cause a number of issues, including noise and “false” spectral structures, which may be easily confused with spontaneous Raman peaks. One approach to overcome these limitations is Shifted Excitation Raman Difference Spectroscopy (SERDS) (Shreve *et al.*, 1992). The combination of the SERDS method and hydrophobin mutants proved that Raman signals from *A. nidulans* originated from dye molecules in the cell wall (Han *et al.*, 2020).

In addition to *A. nidulans*, the Raman spectra of the pathogenic fungi *A. fumigatus* and *Cryptococcus neoformans* and their melanin biosynthetic mutants were analyzed in a similar method (Strycker *et al.*, 2021). Comparisons of Raman spectra allowed for the approximate classification of melanin biosynthetic pathways. Melanin is an important defense mechanism against chemical stresses, such as reactive oxygen species (ROS), and plays a critical role in the virulence of many fungi (Jacobson and Tinnell, 1993; Smith and Casadevall, 2019).

## Conclusion

Fluorescence imaging, with its high specificity and spatiotemporal resolution, is suitable for elucidating molecular mechanisms. Raman spectroscopic imaging is label-free and simultaneously provides spatially resolved information on the relative amounts and chemical states of multiple intracellular components. The strengths of each method are complementary, and the combination of these two methods will provide a more detailed understanding of the chemical properties of hyphae and heterogeneity within the mycelium. The visualization of secondary metabolites *in vivo*, which is impossible with fluorescence imaging, is now feasible for the first time with Raman microspectroscopy and imaging. The combination of mass spectrometry and genetic manipulation is expected to greatly expand the possibilities of applications, such as screening the microbial production of bioactive substances. A reevaluation of the physiological state of various mutant strains obtained to date using the new method may reveal chemical properties that have yet to be identified. Furthermore, this combination will be applied to interactions between filamentous fungi and others, such as fungi and bacteria (Abeysinghe *et al.*, 2020), fungi and plants, and also environmental samples that are difficult to genetically manipulate (Li *et al.*, 2012), through chemical communication and/or metabolic interactions.

## Citation

Shigeto, S., and Takeshita, N. (2022) Raman Micro­spectroscopy and Imaging of Filamentous Fungi. *Microbes Environ ***37**: ME22006.

https://doi.org/10.1264/jsme2.ME22006
